# SMART Markers: collecting patient-generated health data as a standardized property of health information technology

**DOI:** 10.1038/s41746-020-0218-6

**Published:** 2020-01-23

**Authors:** Raheel Sayeed, Daniel Gottlieb, Kenneth D. Mandl

**Affiliations:** 1 Computational Health Informatics Program, Boston Children’s Hospital, Boston, MA USA; 2 Department of Pediatrics, Harvard Medical School, Boston, MA USA; 3 Department of Biomedical Informatics, Harvard Medical School, Boston, MA USA

**Keywords:** Translational research, Health care

## Abstract

A patient-centered health system needs precise computable measurements to derive value. While validated patient-reported outcomes (PROs) are increasingly used in trials, their adoption in care remains limited and generally separated from the medical record. Further, absence of systematic processes for patient-led data submission excludes valuable data from digital devices that can potentially aid in contextualizing health status. With prior experience in developing apps for the Patient-Reported Outcomes Measurement Information System (PROMIS), we sought to make collecting patient-generated health data (PGHD) a fundamental property of health information technology at scale, and in an interoperable, standards-compliant fashion. We build upon the open SMART on FHIR (Fast Health Interoperability Resources) specification to create SMART Markers—a mobile device software framework encapsulating functionality needed for rapid deployment of both patient- and practitioner-facing PGHD apps. We refactored previously developed PROMIS apps to use SMART Markers for handling PGHD-request creation, on-device administration, and generation of a variety of PGHD types and submission of results to a FHIR server. Validation and conformance tests were performed on the generated output and app-reusability was demonstrated across two demo servers. App developers can import SMART Markers into their existing or new apps to readily leverage an interoperable PGHD capturing functionality out of the box, without having to reinvent the wheel. Our approach enables the creation of health system integrated, context-specific experiences for both patients and practitioners.

## Introduction

Over the past decade, as the health system has grappled with implementation of provider-centric electronic health record systems (EHR), the patient voice has largely been omitted from the corpus of routinely collected digital health information.^1^ Thus, the clinical and research enterprises are beginning to incorporate patient-generated health data (PGHD) to define endpoints for treatment, for trials, and to measure value.^2–5^ The 21st Century Cures Act of 2016 includes provisions under Title III to formulate a more robust framework for drug development and product labeling that specifically accounts “patient experience data”, including patient-reported outcomes measures (PROs), to capture not only the disease burden, but also treatment burden. The Food and Drug Administration (FDA) is directed to issue guidance for collecting “robust and meaningful patient or caregiver input”^6^ as part of real-world evidence to aid the drug approval processes establishing a path for routine collection of patient-generated data in clinical trials.^7^

EHRs currently offer access to patients of a subset of their health data through tethered ‘Patient Portals’ and may offer secure electronic messaging. None yet accept data generated by patient’s connected devices (e.g., smartphones and wearables). While some vendors have begun to offer limited support for select PROs, their approaches are generally limited, disparate, non-standardized, and rarely incorporated into routine clinical workflows. Moreover, the lack of intuitive user-interfaces and workflows for patients to specifically generate and submit data likely limit patient engagement.

The SMART on FHIR application programming interface (API) specification fosters an ecosystem of health apps^8^ using HL7’s (Health Level Seven International) Fast Health Interoperability Resources (FHIR)^9^ as the sole data model. SMART has achieved widespread industry adoption across major EHR products and cloud products,^10^ with both practitioner- and patient-facing apps such as Apple’s iOS Health app.^11^ Implementation of these standards is a required component of certified health information technology under the proposed rules^12^ following the 21st Century Cures Act API provisions.

In prior work,^13^ we developed SMART on FHIR apps^14^ to order, administer and review “Patient-Reported Outcomes Measurement Information System” (PROMIS)^15^ instruments. The initial version used a custom API^16^ for instrument discovery and computer adaptive session administration. In so doing, it became clear that there were key functions which could be encapsulated in a common framework library that would enable app developers to readily create use-case specific PRO apps, and further, to harness mobile device-generated personal health data.

In response, we sought to leverage the SMART on FHIR^17^ API to develop SMART Markers, a framework for making patient-generated health data an integral part of both, routine care and research at scale using interoperable standards. The framework is modeled on key actions that build capacity for health systems to enable (1) PGHD requesting as an institution-wide orderable service, available to all its practitioners and (2) patients to generate data for the requested instruments on their personal devices and submit to their health systems in a seamless electronic workflow that is reviewable by practitioners at point of care.

## Results

**Fig. 1:**
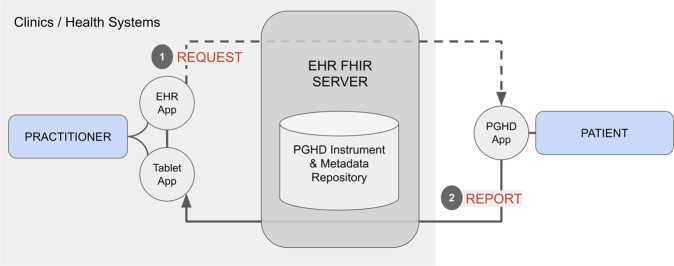
PGHD apps connected through an EHR FHIR server. (1) Practitioner-facing apps dispatching PGHD requests through an EHR app or a Tablet app used in clinics or health systems. (2) Patient-facing apps for generating and reporting practitioner requested PGHD.

SMART Markers was designed principally to enable *request* and *report* actions through health system integrated apps as illustrated in Fig. 1. These actions enable practitioners to dispatch data submission *requests* to the patients from point of care (EHR App, Tablet App), and patients to respond to those requests by generated data from apps on their personal devices (PGHD App) or an institution supplied device. The resulting data from these actions are stored and mediated through the health system’s FHIR server (EHR FHIR server). The initial version supports a diverse set of PGHD types, including PROs, digital markers and activity measures.

SMART Markers changes the paradigm from creation of one-off apps to a common approach; developers use its framework bundle wherein essential PGHD workflows (requesting, capturing, and report submission methods) are encapsulated in a single common unit.

### FHIR encoded PGHD instruments and metadata

**Fig. 2:**
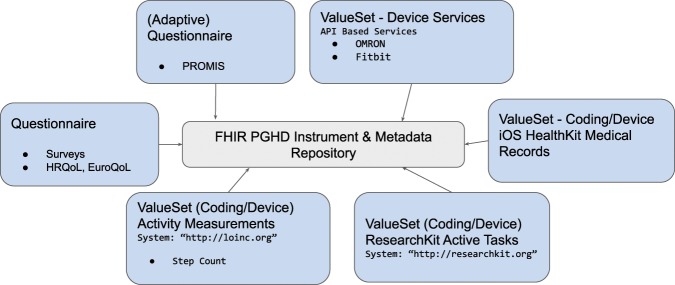
FHIR-based repository storing codified identifiers for different types of PGHD instruments.

We envision the EHR FHIR server or a health-system designated FHIR-server based repository to host instruments or their metadata approved for hospital-wide use in conformance with FHIR. A variety of instrument types can be encoded as shown in Fig. 2. As a result, apps using SMART Markers can compile a list of available instruments from this repository.

Survey-type instruments are encoded as FHIR Questionnaire resources. The FHIR Adaptive Questionnaire profile^18^ specifies an API for administering computer adaptive surveys, including through third-party service providers (e.g., PROMIS). Summarized versions of such instruments can be hosted in the repository as FHIR Questionnaire resources with an HL7 defined extension^19^ indicating its adaptive nature, and a uniform resource locator pointing to the service provider for initiating the adaptive Q&A session. This enables health systems to add third party adaptive services into their pre-approved pool of instruments.

To precisely identify medical devices (glucometers, blood pressure monitors), wearables or apps that generate health data; their data-type or software identifiers are encoded as FHIR ValueSet resources using appropriate ontologies. Table 1 lists the distinct PGHD instrument categories and devices supported in this iteration and the corresponding outcome FHIR resources.

**Table 1:** FHIR Conformance of PGHD instrument types.

Instrument Category	Sub Type	FHIR Encoding	Instrument	FHIR Result Resource
Surveys	Static^36^	Questionnaire	Social determinants, quality of life surveys, patient-reported outcomes	QuestionnaireResponse
	Dynamic	Questionnaire (Adaptive IG)	PROMIS	QuestionnaireResponse (Adaptive IG)
Device recorded	Activity	Coding	Step count	Observation
Connected web repositories^22^	Vital Signs	Coding	OMRON Blood Pressure	Observation
Active tasks	Motor	Coding	Range of motion (knee, shoulder)	Observation (Angle)
			Tapping speed	Observation DocumentReference
			9 Peg hole test	Observation DocumentReference
	Cognition	Coding	Paced serial addition test	Observation DocumentReference
			Tower of Hanoi	Observation (Boolean)
			Stroop test	Observation (Duration)
			Spatial Memory Span	Observation (Score)
	Vision	Coding	Amsler grid	Observation Media (Image)
FHIR Data (Apple Health App)				FHIR R4

Active task measurements are supported by modules in ResearchKit.^24^

### Dispatching PGHD requests at the point of care

**Fig. 3:**
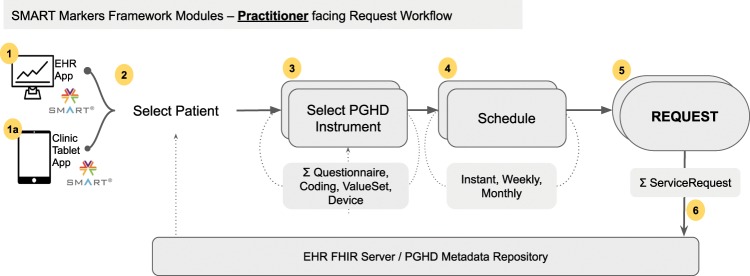
SMART Markers framework modules for practitioner-facing apps interacting with a SMART enabled EHR FHIR Server. Data Flow is shown: The practitioner: (1,1a) launches the app at point of care. (2) selects a patient. (3) the app compiles a list of PGHD instruments from the EHR/PGHD FHIR repository. (4) the practitioner selects a schedule for the request. (5) A FHIR ServiceRequest is generated per instrument. (6) Requests are submitted to FHIR Server.

The SMART Markers framework includes a requesting module for practitioner-facing apps to dispatch a FHIR ServiceRequest resource to the selected patient, illustrated in Fig. 3.

**Fig. 4:**
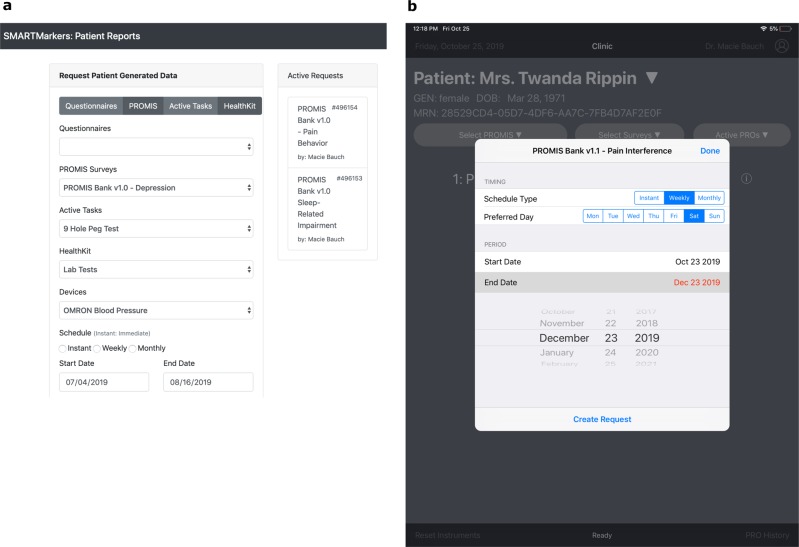
Practitioner apps at point of care. **a** EHR app running within the context of an EHR session for dispatching PGHD Instrument requests. **b** Practitioner Tablet app to dispatch or administer instrument sessions for ambulatory settings.

Each request is embedded with a reference to the instrument compatible FHIR resource. Surveys that are encoded as FHIR Questionnaire resources are referenced through an HL7 defined extension^20^ to specifically identify the questionnaire. Other PGHD types can be referenced in the request through its instrument-specific ontological code. Optionally, the request can be associated with an allotted time frame or recurring schedule during which the framework enables the PGHD session to be administered. The current iteration supports instant or period-bound weekly or monthly time frames. Figure 4 is a screenshot of a sample app interfaces.

### Report generation and submission

**Fig. 5:**
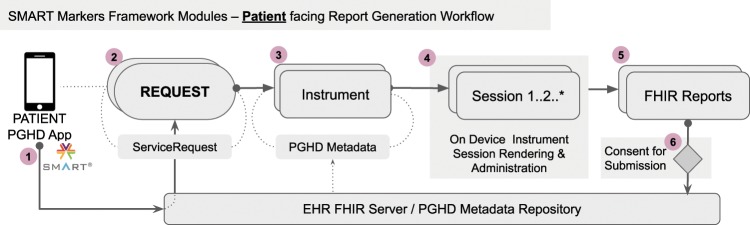
SMART Markers framework modules for patient-facing app interacting with SMART enabled EHR FHIR server. Data Flow is shown: Patient: (1). Launches a PGHD app connected to the EHR FHIR Server. (2): After logging-in, fetches PGHD related requests. (3): App verifies availability of instrument identified in each request. (4): Begin user session on device and administer the instrument. (5): Generation of FHIR formatted results. (6): User consent for submission of reports to FHIR server.

For a patient-facing app, the framework constructs a list of PGHD requests from the FHIR server and resolves the PGHD instrument embedded in each request along with the associated due date or a repeating schedule (Fig. 5).

Each FHIR Questionnaire resource is transformed into ResearchKit steps to produce an in-app Q&A session which captures patient responses and generates a resultant FHIR resource–QuestionnaireResponse. Adaptive Questionnaire sessions are created similarly, with the framework recognizing and handling its dynamic API calls in the background but rendering the same core user experience. This is enabled by standardized FHIR “next-q” API operation specified by the PRO implementation guide^21^ from the Office of National Coordinator for Health Information Technology (ONC).

Moreover, the framework’s protocol-oriented approach establishes a foundation for additional data aggregating or generating instruments (other than questionnaires) within the session protocol. For example, OMRON devices can store recorded blood pressure data in their cloud store which is accessible to developers via their APIs.^22^ The SMART Markers framework includes an OMRON module that allows patients to fetch their data from the OMRON cloud, encode it into a FHIR Observation resource and submit it to a health system– *all within a survey-style session*. Similarly, modules can be added to fetch user data from other third-party health data repositories like the Fitbit.^23^

Apple ResearchKit-based active tasks^24^ use device sensors to measure motor activity, cognitive function and vision as listed in Table 1. After completing these tasks on device, the framework transforms results into FHIR resources that best reflect the task’s data type. Further, smartphone-produced activity data (e.g., step count) are harmonized and encoded in a FHIR resource.

**Fig. 6:**
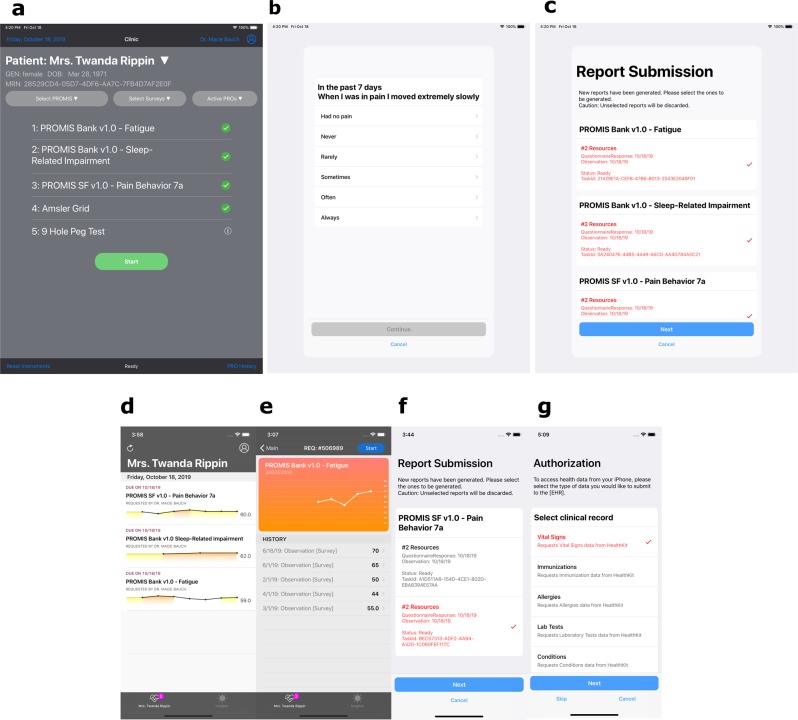
PGHD application screenshots. Practitioner-facing app **a** dashboard view for PGHD selection. **b** PROMIS session rendered by ResearchKit. **c** PGHD report submission step. Patient-app **d** main view listing all requests. **e** Visualization of historical record. **f** Report submission step. **g** Apple Health FHIR data capturing task module.

Specifically, for Apple iOS’s Health app, SMART Markers includes a task handler (Fig. 6g) to request access to the user’s medical record stored in iPhone’s secure on-device health data store– HealthKit. The resultant DSTU2 based FHIR data are mapped into R4 version following the US Core implementation guide.^25^

Upon successful completion of each of the instrument sessions, the framework generated reports, packaged as a set of FHIR Bundle resources, are presented to a reporting module which seeks user confirmation before tagging the FHIR resources with the patient and requester reference and finally submitting to the health system’s FHIR server.

### Implementing SMART on FHIR PRO apps with SMART Markers

Previously developed PROMIS apps were successfully refactored to use the SMART Markers framework^26^ (Fig. 6). We implemented the FHIR compliant endpoint of the Assessment Center (AC)—the external computer adaptive service provider for PROMIS. The framework’s PROMIS server module is first initialized with “Basic” authentication credentials obtained from AC which is then used to fetch a complete list of available instruments and presented to the end user for selection. Upon initiation of an instrument session, the framework compiles all Q&A items of the selected instruments and generates ResearchKit steps as described in the previous section. In addition, we expanded the apps with additional PGHD instrument types supported by SMART Markers.

All generated PGHD were validated for basic structural integrity and cardinality as per FHIR R4 in JSON format. We verified user input in survey sessions by comparing FHIR output with the renderer-derived (ResearchKit) task result. Further, the FHIR QuestionnaireResponse resource was validated to reflect its matching FHIR Questionnaire. To promote app-reusability, both apps were made compliant with and shown to be functional on the demo SMART on FHIR servers. For adaptive PROMIS instruments, a universally unique identifier was generated and associated with their task handlers to administer adaptive testing. This identifier was sent to AC’s stateless FHIR API to initiate a Q&A session. No patient information was sent to the server other than the patient responses during or after completion. This is verifiable by the open source code of the framework making the API request call and enforced in code to only use the randomly generated identifier. The resulting output was adjusted to FHIR R4 and successfully posted to the demo servers through the reporting module described above.

## Discussion

SMART Markers is a framework for integrating PGHD and its various subtypes—PROs, digital biomarkers, and sensor data from connected devices with a patient-centric reporting process bringing data to point of care.

Even as the health system gains experience with patient-reported outcomes, the patient voice is not yet incorporated in a standardized way. In 2004, the National Institutes of Health launched PROMIS to produce validated, nationally scalable instruments^15^ using psychometric methods to create computer adaptive tests, wherein a question item is informed by the preceding answer and can be retrieved electronically through an API. While enabling PROs as a clinically orderable service is a relatively recent endeavor, several leading institutions have incorporated PROs in routine care with varied implementation strategies.^27^ For example, Partners Healthcare has electronically captured over 1.2 million responses by engaging with the EHR to natively integrate PROs.^28^ The University of Rochester developed its own custom-built solution and collected over 1.1 million responses using iPads in the waiting rooms.^29^ Similarly, the University of Utah developed its “mEVAL” PRO service that is tightly connected to its enterprise data warehouse; with staff dispatching PRO requests to patients via email or administer on tablets onsite.^30^ The University of California & University of Michigan co-developed the “My GI Health” project as a dedicated third-party PRO service^27^ serving site and condition specific needs. There have been some efforts toward open source PRO capturing software. For example, the FDA’s MyStudies app^31^ is a research focused digital platform which allows constructing surveys in its “web configuration portal”. However, because both the surveys and the responses are transacted and stored in its custom format, the app does not leverage standards-based data exchange through interoperability.

Capturing PGHD has been limited to PROs, has generally involved custom EHR integrations using non-standardized data models or a workflow entirely outside the EHR (as is the case in trials). Further, the PRO systems are not effectively integrated with contextualized information needed for clinical decision support. While surveys are sometimes codified in LOINC,^32^ a lack of standardized ontology for patient-generated digital data constrains syntactic interoperability of measurements among institutions, which in turn hinders the generalizability of quality measurements at a population scale.

SMART Markers’ strict adherence to standards where FHIR remains the sole transacted element, renders it reusable across a broad array of use cases without incurring significant, or frequent enhancements to the EHR vendor products. Moreover, as a framework for mobile devices, it achieves three important goals. Firstly, SMART Markers can be readily imported into other apps, enabling turnkey usage of embedded functionality. Secondly, context-dependent apps can be built using the same framework to render these apps universally deployable across standards-compliant systems. Thirdly, defining a minimum set of methods in an embeddable framework used by a number of apps downstream greatly simplifies app maintenance and retains consistency of user experience. Notably, both patient-facing and practitioner-facing apps can be built using the same framework. This abstracted functionality from apps enables easy customizations involving the FHIR resources or the client-side framework. In contrast to web-apps, device-native apps (for iOS or Android) offer more granular control over the app life cycle which is crucial when handling device APIs to access sensor data. This is demonstrated with the framework’s support of active-tasks to deduce shoulder or knee “range of motion” that uses the device’s accelerometers. In addition, native apps do not require a webhost as an intermediary gateway to facilitate data capture and reporting. Our focus is to enhance the patient’s reporting experience and support a diverse array of instrument types.

With this approach, we seek to empower patients with connected apps to proactively participate and control the flow of their data into the health system which is implicit in our reporting model. As smartphones continue to evolve and become secure personal health repositories, our framework demonstrates the capacity to facilitate such patient-led submission of PGHD from a variety of instruments, wearables, and digital devices through a familiar clinical construct.^33^ In addition, SMART Markers can be adapted to enable novel use-cases with onsite or in-clinic kiosk setups initiating survey sessions for not only PROs but also to submit health data stored in growing list of web-based personal health repositories (e.g., Omron)—at the point of care, using FHIR.

As PGHD capturing practices becomes routine, it will be important to avoid overwhelming patients with data requests. Effective strategies can be taken to avoid patient fatigue and maintain compliance. Our model is designed to avoid duplicated requests and promote judicious use from across clinics and departments, as all active requests and incoming PGHD for a patient are reviewable by all practitioners at all times. In turn, our hope is that patients become more engaged knowing that their valuable data are not lost in obscure databases, but instead are promptly available at the point of care for better informed and shared decision-making. Having a streamlined reporting process endorsed by health systems, using modern tools with fluid interfaces may further improve patient engagement.

There remain important dependencies on other ecosystem components. For example, automated PGHD capture from wearables and connected devices, with device provenance, necessitates further commitment from device manufacturers to adopt interoperable standards. A notable effort is the proposed “Personal Health Device” implementation guide^34^ that defines a minimum set of variables needed to represent patient’s personal devices being used as gateways in FHIR Device resource along with its codable concept. Manufacturers conforming to such standards with accessible APIs can expedite software implementations and further encourage sharing practices.

By open sourcing all components through GitHub (https://github.com/smartmarkers)– the Swift framework for mobile devices, including an EHR integrated app written in Python, we plan to inform ongoing development with input from the community.

SMART Markers is evolving and while the initial version is for iOS devices, we intend to support substitutability^1^ by creating an Android app built on a similar standardized model of request and reporting. The Android app will be on be on par with its iOS counterpart within the constraints of devices and on-board sensors. For our cross-platform strategy, we are funded by the ONC to develop SMART Markers in React-Native language. Indeed, we are building the Android analog with a similar patient-centric approach. Further exploration will include using ResearchStack^35^ as the ResearchKit counterpart for consistency on Android devices. Broader support of PGHD and devices, patient-wise survey recommendation engines, subscriptions (to be notified of PGHD-related events) in the EHR—are all potentially addressable as interoperable components with open standards.

As an open source project, our intention is to enable healthcare institutions to integrate the framework into their existing apps or deploy customized versions of the reference apps in their context-specific use-cases. As has been our approach in SMART on FHIR and other SMART projects, we hope, through community experience and feedback, to test our specifications against real world experience. It will be important to design evaluations of app usability, patient use and adherence, and to compare data obtained through SMART Markers to those obtained by current data collection practices.

## Methods

### Collection scenarios

Within a typical health system, there are multiple contexts for practitioners to facilitate PGHD collection. Outpatient clinics and waiting rooms remain the frequent sites for such collection under practitioner supervision. Inpatients are often surveyed as needed, for example, to assess pre- and post-surgery status. In this context, ambulatory EHR connected apps for mobile tablets can be used to collect PGHD onsite by administering survey instruments or dispatching PGHD requests to patients. Similarly, apps launched in the context of an EHR workspace can surface such functionality at the point of care.

Patients may be much more likely to participate in producing PGHD if offered a seamless pathway to do so in their natural environments beyond hospitals or in clinical settings. In this scenario, the patients respond to the PGHD requests by participating in data generating sessions on smartphones or tablets. In both cases, the data are submitted to the EHR FHIR Server.

### Standards based integration architecture

We use SMART on FHIR as the sole user authorization mechanism for apps to securely connect to health systems where they solely interact with FHIR endpoints for reading and writing clinical data. Optionally, a health system may provision an additional FHIR server for hosting PGHD related metadata, for example, instruments, metadata and/or incoming patient reports.

A secondary FHIR store may be necessary for some EHR-linked FHIR environments that primarily focus on accessing EHR derived data and do not incorporate robust support for writing clinical information.

The initial iteration is programmed with the *Swift* programming language for Apple’s iOS operating system and uses *Python* for web components, building on existing open source SMART on FHIR client and server libraries.

### PGHD instrument & session rendering

As test instruments, a variety of PGHD instrument types were selected (listed in Table 1). Survey examples encoded as FHIR Questionnaire resources were obtained from HL7.^36^ PROMIS’ FHIR API was used to test computer adaptive testing. Developer credentials were obtained from Omron to fetch blood pressure data from its web repositories.

Apple’s open source mobile research framework, ResearchKit,^37^ is used as the interface layer to administer on-device PGHD sessions and maintain a cohesive user-experience for patients to proactively respond and submit the generated data to the health system and also retain cross compatibility with other research frameworks.^38^

### Validation and scalability

To validate SMART Markers, we refactored two previously developed^39^ PROMIS apps (for practitioners and patients) and adapted them to use the framework as their underlying engine. We tested the reusability of the apps across two demonstration servers provided by SMART Health IT^40^ and HSPC^41^ sandboxes. We further validated the FHIR conformance of the framework generated PGHD using publicly available HL7 validation tools.

### Reporting summary

Further information on research design is available in the Nature Research Reporting Summary linked to this article.

## Supplementary information

Reporting Summary

## Data Availability

Sample FHIR Questionnaires can be found at https://www.hl7.org/fhir/questionnaire-examples.html. Access to PROMIS Adaptive FHIR instruments (API keys) were sought from https://www.assessmentcenter.net.

## Code availability

SMART Markers Framework code, documentation, including reference practitioner and patient apps can be found at https://github.com/smartmarkers released with Apache 2.0 open source license.

## References

[1] Mandl KD, Kohane IS (2009). No small change for the health information economy. N. Engl. J. Med..

[2] Basch E (2017). Patient-reported outcomes—harnessing patients’ voices to improve clinical care. N. Engl. J. Med..

[3] Lavallee DC (2016). Incorporating patient-reported outcomes into health care to engage patients and enhance care. Health Aff..

[4] Marshall S, Haywood K, Fitzpatrick R (2006). Impact of patient-reported outcome measures on routine practice: a structured review. J. Eval. Clin. Pract..

[5] NBT-Editorial. (2019). Getting real with wearable data. Nat. Biotechnol..

[6] Center for Drug Evaluation & Research. CDER’s Patient-focused drug development. *U.S. Food and Drug Administration* https://www.fda.gov/drugs/development-approval-process-drugs/cder-patient-focused-drug-development (2019).

[7] Goble JA (2018). The potential effect of the 21st century cures act on drug development. J. Managed Care Specialty Pharm..

[8] Mandl KD, Mandel JC, Kohane IS (2015). Driving innovation in health systems through an apps-based information economy. Cell Syst..

[9] Welcome to the HL7 FHIR Foundation. http://fhir.org.

[10] Patient-Led Data Sharing — A new paradigm for electronic health data. *NEJM Catalyst* https://catalyst.nejm.org/patient-led-health-data-paradigm/ (2018).

[11] Apple announces solution bringing health records to iPhone. *Apple Newsroom* https://www.apple.com/newsroom/2018/01/apple-announces-effortless-solution-bringing-health-records-to-iPhone/.

[12] Notice of proposed rulemaking to improve the interoperability of health information | HealthIT.gov. https://www.healthit.gov/topic/laws-regulation-and-policy/notice-proposed-rulemaking-improve-interoperability-health.

[13] $6.3 million NIH grant to spur use of patient reported outcomes in EHRs. *Healthcare IT News*. https://www.healthcareitnews.com/news/63-million-nih-grant-spur-use-patient-reported-outcomes-ehrs (2016).

[14] AMIA-EASIPRO. System demonstration: integration of patient reported outcomes with electronic health records—the EASI-PRO Project. https://knowledge.amia.org/67852-amia-1.4259402/t008-1.4262115/t008-1.4262116/2976057-1.4262126/2975528-1.4262123?qr=1.

[15] Cella D (2007). The Patient-Reported Outcomes Measurement Information System (PROMIS): progress of an NIH Roadmap cooperative group during its first two years. Med. Care.

[16] Assessment Center. http://www.assessmentcenter.net.

[17] Mandel JC, Kreda DA, Mandl KD, Kohane IS, Ramoni RB (2016). SMART on FHIR: a standards-based, interoperable apps platform for electronic health records. J. Am. Med. Inform. Assoc..

[18] HL7.FHIR.UV.SDC\Adaptive forms - FHIR v4.0.0. http://build.fhir.org/ig/HL7/sdc/adaptive.html.

[19] SDC\Adaptive Questionnaire - FHIR v3.5.0. http://hl7.org/fhir/uv/sdc/2018Sep/sdc-questionnaire-adapt.html.

[20] Extension: questionnaireRequest - FHIR v4.0.0. http://hl7.org/fhir/extension-servicerequest-questionnairerequest.html.

[21] Office of the National Coordiantor. Patient_Reported_Outcomes. http://hl7.org/fhir/us/patient-reported-outcomes/2018Sep/pro-overview.html.

[22] Omron API for Developers | Omron Healthcare. *Healthcare Wellness & Healthcare Products*. https://omronhealthcare.com/api/.

[23] Fitbit Development: Fitbit SDK. https://dev.fitbit.com.

[24] ResearchKit-ActiveTasks. Active Tasks. http://researchkit.org/docs/docs/ActiveTasks/ActiveTasks.html.

[25] HL7 International-US Realm Steering Committee. USCore. http://hl7.org/fhir/us/core/2019Sep/r2-r4-guidance.html.

[26] easipro-clinic-pghd-ios. https://github.com/SMARTMarkers/easipro-clinic-pghd-ios.

[27] Jensen RE (2015). The role of technical advances in the adoption and integration of patient-reported outcomes in clinical care. Med. Care.

[28] Rotenstein LS, Huckman RS, Wagle NW (2017). Making patients and doctors happier—the potential of patient-reported outcomes. N. Engl. J. Med..

[29] Papuga MO (2018). Large-scale clinical implementation of PROMIS computer adaptive testing with direct incorporation into the electronic medical record. Health Syst. (Basingstoke).

[30] Biber J (2017). Patient reported outcomes - experiences with implementation in a University Health Care setting. J. Patient Rep. Outcomes.

[31] Office of the Commissioner. New real world evidence digital tool from FDA, MyStudies app. *U.S. Food and Drug Administration* https://www.fda.gov/NewsEvents/Newsroom/FDAInBrief/ucm625228.htm (2019).

[32] LOINC. Survey instruments Archive - LOINC. https://loinc.org/panels/category/survey-instruments/.

[33] Kovalchick C (2017). Can composite digital monitoring biomarkers come of age? A framework for utilization. J. Clin. Transl. Sci..

[34] Continua personal health device data implementation guide. http://hl7.org/fhir/uv/phd/2018Jan/index.html.

[35] ResearchStack. http://researchstack.org.

[36] Questionnaire - FHIR v4.0.1. https://www.hl7.org/fhir/questionnaire-examples.html.

[37] Sanchez-Saez, R. et al. Researchkit/Researchkit: Researchkit 1.5.3. (2017).

[38] Pfiffner Pascal B., Pinyol Isaac, Natter Marc D., Mandl Kenneth D. (2016). C3-PRO: Connecting ResearchKit to the Health System Using i2b2 and FHIR. PLOS ONE.

[39] EASIPRO – Seamless integration of patient-reported outcome measures in electronic health records. https://sites.northwestern.edu/easipro/.

[40] SMART App Launcher. https://launch.smarthealthit.org.

[41] HSPC | Build Amazing Apps. *hspc-developers*. https://www.developers.hspconsortium.org/build.

